# A Meta-Analysis on the Efficacy and Safety of Bacterial Lysates in Chronic Obstructive Pulmonary Disease

**DOI:** 10.3389/fmed.2022.877124

**Published:** 2022-06-09

**Authors:** Yongkang Huang, Yongjian Pei, Yajuan Qian, Zhen Yao, Chen Chen, Juan Du, Minhua Shi, Tong Zhou

**Affiliations:** ^1^Department of Respiratory and Critical Care Medicine, The Second Affiliated Hospital of Soochow University, Suzhou, China; ^2^Department of Respiratory and Critical Care Medicine, The First Affiliated Hospital of Soochow University, Suzhou, China

**Keywords:** chronic obstructive pulmonary disease, bacteria lysates, OM-85, Ismigen, efficacy, safety

## Abstract

**Background:**

Chronic obstructive pulmonary disease (COPD) is a common and frequently encountered disease of respiratory apparatus and is vulnerable to infection. Increasing studies reveal that bacterial lysates play an encouraging role in preventing exacerbations in these patients. We here investigated the efficacy and safety of bacterial lysates in COPD.

**Methods:**

We performed systematic research on PubMed, EMBASE, the Cochrane Library (CENTRAL), and Web of Science by using the keywords and their synonyms for studies published before January 11, 2022. Two researchers screened the studies of literature independently according to the inclusion and exclusion criteria and extracted data from the included studies. Another two researchers assessed the risk of bias of each included using the Cochrane risk-of-bias tool. Meta-analysis was conducted using R (version 4.1.1, The R Foundation for Statistical Computing) and Review Manager (version 5.4.0, The Cochrane Collaboration).

**Results:**

A total of 12 studies were included in this meta-analysis, and the pooled results showed that bacterial lysates were effective to reduce exacerbation rate (overall: relative risk [RR] = 0.83, 95% confidence interval [CI] 0.72–0.96; alkaline bacterial lysate subgroup [OM-85]: RR = 0.87, 95% CI 0.77–0.98; mechanical bacterial lysate subgroup [Ismigen]: RR = 0.70, 95% CI 0.41–1.20) and mean number of exacerbations (overall: MD = −0.42, 95% CI −0.75 to −0.08; alkaline bacterial lysate subgroup [OM-85]: MD = −0.72, 95% CI −1.35 to −0.09; mechanical bacterial lysate subgroup [Ismigen]: MD = −0.02, 95% CI −0.21 to 0.17). Bacterial lysates were also found beneficial in alleviating symptoms. The side effects were acceptable and slight.

**Conclusion:**

Bacterial lysates can benefit patients with COPD by reducing exacerbations and alleviating symptoms. OM-85 is the preferable product based on the existing evidence. Further studies are needed to validate these findings.

**Systematic Review Registration:**

[www.crd.york.ac.uk/prospero/], identifier [CRD42022299420].

## Background

Chronic obstructive pulmonary disease (COPD) is a preventable, treatable chronic respiratory disease that results from the airway or alveolar abnormalities and is one of the main causes of death globally (1). Furthermore, the population with COPD will increase with aging and continued exposure to risk factors (2). The goal of COPD treatment is to relieve symptoms and reduce exacerbations, improve the quality of life, and decelerate progression. Bronchodilators are the main treatment in most COPD guidelines. However, acute exacerbations still occur despite an optimal bronchodilator regimen. Moreover, older adults who have low economic and education levels account for a large proportion of those with COPD (3). For these people, an inhaler with a bronchodilator might not be the most acceptable and economical choice. New treatments are still needed.

Bacterial lysates are antigen-containing products derived from several common bacteria that infect humans, including *Staphylococcus aureus*, *Staphylococcus pneumoniae*, *Staphylococcus pyogenes*, and *Haemophilus influenzae*. Based on the preparation method, they are classified as alkaline or mechanical bacterial lysates. Commercial products of both types are available; OM-85 and Luivac are alkaline bacterial lysates and Ismigen is a mechanical bacterial lysate. Studies have shown that bacterial lysates reduce infections and antibiotic use (4) and are cost-effective (5, 6). A systematic review and meta-analysis of COPD found that OM-85 reduced the exacerbation rate and need for antibiotics (7). However, the study evaluated a single alkaline bacterial lysate and it was published nearly 7 years ago. As several important clinical trials have been conducted since then (8–10), an updated, comprehensive meta-analysis is warranted.

## Methods

This systematic review and meta-analysis evaluated the efficacy and safety of bacterial lysates in COPD based on the latest evidence. The study was registered at PROSPERO (registration number: CRD42022299420) and followed the Preferred Reporting Items for Systematic Review and Meta-Analysis (PRISMA) guidelines (11).

### Inclusion and Exclusion Criteria

#### Participants

The participants were adults (age > 16 years) suffering from COPD, characterized by persistent respiratory symptoms and airflow limitation defined by a post-bronchodilator FEV_1_/FVC ratio < 0.70.

#### Type of Intervention and Comparator

The intervention was a bacterial lysate, including OM-85, Ismigen, MV130, Luivac LW50020, Lantigen, and Ribomunyl. The comparator was a placebo or blank.

#### Type of Studies

A randomized controlled trial or cohort study.

#### Outcomes

The primary efficacy outcome was the effect of the bacterial lysate on the rate and frequency of acute exacerbations; secondary efficacy outcomes included respiratory tract infections, hospitalization rate, and symptom improvement (sputum, severity of dyspnea, frequency of cough, and fever). Safety was evaluated based on adverse effects associated with the therapy, including symptoms and the overall rate of adverse events.

The studies reviewed met the above criteria, and studies were excluded if they used the same or overlapping data by the same authors or lacked any one of the predefined outcomes.

### Literature Search Strategy

Data were retrieved from PubMed, EMBASE, the Cochrane Central Register of Controlled Trials (CENTRAL), and Web of Science by an experienced researcher using keywords and their synonyms for studies published before January 11, 2022. Search terms were modified to be commensurate with each database’s index terms, such as Medical Subject Heading (MeSH) in PubMed and Emtree in EMBASE. Relevant citations listed the references in each included study were also screened for eligibility to minimize retrieval bias. Supplementary Appendix 1 lists the search terms.

### Study Selection

Two trained researchers selected studies independently. Duplicates were identified and removed by comparing the author, title, and publication year. The remaining studies were scanned by the title and abstract, and irrelevant studies were omitted, while the full texts of the others were read. Studies listed in the references of included studies were also screened for eligibility.

### Quality Assessment

Two researchers independently assessed the quality of all selected studies. The Cochrane risk-of-bias tool was chosen and included the following items: random sequence generation, allocation concealment, blinding of participants and personnel, blinding of outcome assessment, incomplete outcome data, selective reporting, and other biases (12). When there was a disagreement between the researchers, the study was re-evaluated by a third researcher and determined by the majority.

### Data Extraction

Two researchers extracted the following data from each included study independently: study authors, publication year, country, number of participants, source of cases, type of bacteria lysate, dosages, administration routes, control, outcomes, study period, follow-up, and registration number; participant demographics, smoking history, forced expiratory volume in 1 s/predicted value (FEV_1_/predicted), and concomitant medications and diseases; and pre-established efficacy and safety outcomes. When results were reported at multiple time points, the one nearest 3 months after the last drug dose was chosen. If per-protocol and intention-to-treat analyses were both reported, the results from the intention-to-treat analysis were preferred. Any disagreements between the two researchers were resolved by consensus or cross-checking with a third researcher. If the information of interest was not reported directly, we tried to deduce it from the existing information or obtain it by communicating with the principal investigator by email.

### Statistical Analysis

An independent statistician performed the statistical analysis using Review Manager version 5.4.0 (The Nordic Cochrane Centre, The Cochrane Collaboration) and R version 4.1.1 (The R Foundation for Statistical Computing). For continuous variables, we reported the mean and standard derivation (SD), the mean difference (MD) or standard mean difference (SMD), and 95% confidence interval (CI). When the units used were consistent, MD was preferable; otherwise, SMD was chosen. For dichotomous variables, we reported the frequency or proportion, relative risk (RR), and 95% CI.

The chi-square test and *I*^2^ statistic were used for identifying and measuring heterogeneity. The *P*-value of chi-square test of less than 0.1 and *I*^2^ of more than 50% indicate heterogeneity, and in this case, a Galbraith radial plot was generated, and meta-regression analysis was used to explore the underlying causes of heterogeneity. A random-effects model was applied for the synthesis, and subgroup analysis was performed, if possible. When there was no heterogeneity, a fixed-effects model was preferred. The results of the meta-analysis are shown with forest plots. We also conducted sensitivity analysis and funnel plot analysis if applicable.

## Results

### Study Selection

**FIGURE 1:**
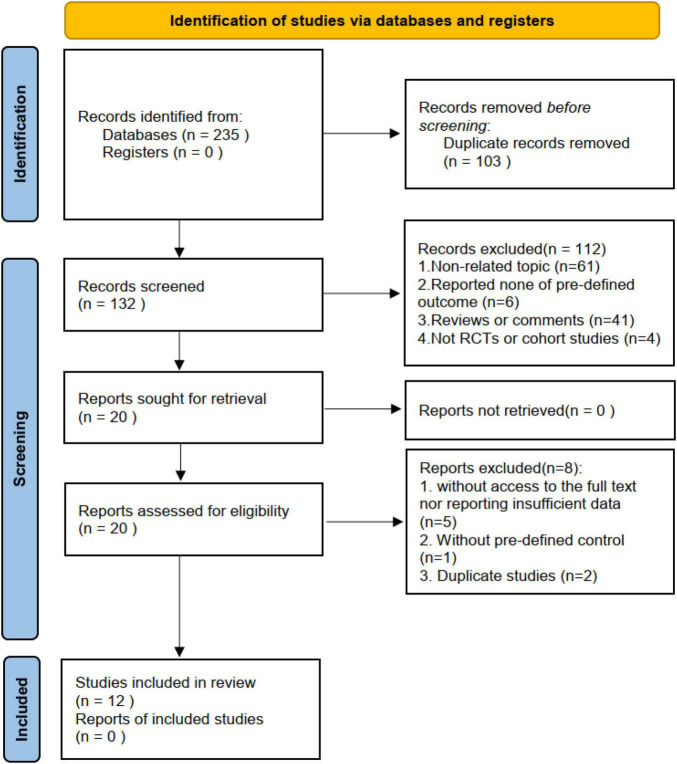
The flow diagram.

The database search yielded 235 studies. Of these, 103 studies were removed as duplicates by comparing the title, publication year, and authors. The abstracts of the remainder were assessed to determine eligibility. This led to the removal of 61 studies that did not involve patients with COPD or did not focus on the clinical application of bacterial lysates, 41 citations of reviews, 6 studies without pre-defined outcomes, and 4 studies that were not a randomized controlled trial or cohort study. The remaining 20 studies were subject to a whole-text review. Subsequently, eight studies were voted out, namely, two duplicate studies; five without access to the full text or reporting insufficient data; and one without a pre-defined control. During the process, we found that the study by Ricci et al. (13) was ancillary to the AIACE study (2), but retained it because it reported complementary data. Ultimately, this meta-analysis included 12 studies, namely, nine randomized controlled trials and three abstracts of randomized controlled trials with sufficient data (8–10, 13–21). Figure 1 summarizes the study selection process.

### Characteristics of the Included Studies and Participants

Of the included studies, seven focused on OM-85 (alkaline bacterial lysate) and five on Ismigen (mechanical bacterial lysate), and studies were conducted in Italy (*n* = 3), China (*n* = 3), Russia (*n* = 1), Canada (*n* = 1), Switzerland and Germany (*n* = 1), Sri Lanka (*n* = 1), and not reported (*n* = 2). Three studies were blank-control, while the others were placebo-control. All studies were parallel randomized controlled trials, with four single-center and eight multicenter studies. Two studies were registered. The majority of cases in the studies were men, in accordance with the real-world population. Most studies allowed flu vaccination. The FEV_1_/predicted ranged from less than 30% to more than 80%. The characteristics of the included studies and participants are summarized in Tables 1, 2, respectively.

**TABLE 1:** The characteristics of the included studies.

Author	Number of participants (bacterial lysate/control)	Major inclusive criteria	Study design		Type of bacteria lysate	Administration way	Dosages and session	
Avdeev et al. (8)	30/30	Patients with frequent exacerbations of COPD (group C and D according to the GOLD classification)	Parallel RCT, multicenter		Ismigen	Sub-lingual	A cycle consisted of sublingual consumption of one tablet per day for 10 consecutive days, followed by 20 days of standard treatment for three consecutive months	
Zeng et al. (14)	78/72	Stable COPD patients	Parallel RCT, single center		OM-85	Oral	A capsule (OM-85 7 mg) or placebo daily for 10 consecutive days per month, for 6 consecutive months	
Tang et al. (9)	192/192	Age 40–75 years, male or female, physician-diagnosed chronic bronchitis, or COPD (for 6 months) suffering from an acute exacerbation according to the GOLD definition at enrollment and an FEV1 between 40 and 70% of the predicted value within the 6 months before enrollment.	Parallel RCT, multicenter		OM-85	Oral	One capsule (OM-85 7 mg) or placebo daily for 10 consecutive days per month, for 3 consecutive months (12 weeks)	
Braido et al. (10)	146/142	A documented diagnosis of moderate, severe or very severe COPD, according to the GOLD 2006	Parellel RCT, multicenter		Ismigen	Sub-lingual	A cycle consisted of sublingual consumption of one tablet per day for 10 consecutive days, followed by 20 days of standard treatment for 3 consecutive months. After 3 months without any PMBL treatment, a second cycle of therapy (as described above) was undertaken. At the end of the second treatment period, a second three-month period without PMBL was observed	
Ricci et al. (13)	12/11	Patients aged 40 years or older with documented moderate, severe or very severe COPD	Parellel RCT, single center		Ismigen	Sub-lingual	A cycle of 90-day treatment wherein the first 10 days of each 30 days 1 tablet of either PMBL or placebo was given. This was followed by a 90-day ‘rest’ period (no treatment).	
Nishantha et al. (15)	24/21	Stable COPD patients with moderately severe or severe disease staging, presenting during May – September 2012	Parallel RCT, single center		Ismigen	Sub-lingual	Daily on the first 10 days of three successive months	
Olivieri (16)	340	Patients aged over 40 years old, with COPD stage II or III, with a history of at least 2 documented AE-COPD in the previous year, and an FEV1 between 30 and 80%	Parallel RCT, multicenter		OM-85	Oral	A capsule daily during month 1, and 1 capsule daily for 10 days in months 3–5.	
Cazzola et al. (17)	33/30	Patients suffering from moderate-to-very severe COPD, who were under regular treatment with salmeterol/fluticasone (SFC) 50/500 mg BID	Parellel RCT, single center		Ismigen	Sub-lingual	one capsule daily the first 10 days of three consecutive months	
Solèr et al. (18)	142/131	Outpatients aged 40 years old of both sexes with a history of chronic bronchitis or mild COPD at the time of an AE	Parallel RCT, multicenter		OM-85	Oral	A capsule of OM-85 or placebo per day for 30 days, followed by three 10-day courses for months 3, 4, and 5	
Li et al. (19)	49/41	Patients with chronic bronchitis complicated with COPD	Parallel RCT, multicenter		OM-85	Oral	A capsule daily for the first 10 days of each month for 3 consecutive months	
Collet et al. (20)	191/190	Patients with a history of heavy smoking and an FEV1 value between 20 and 70% of predicted	Parallel RCT, multicenter		OM-85	Oral	A capsule per day for 30 days followed by a repeat course of 10 consecutive days of therapy per month for 3 months	
Xinogalos et al. (21)	33/29	–	Parellel RCT, multicenter		OM-85	Oral	A capsule (7 mg)/day for 1 month; 1 capsule/10 days for months 3, 4, and 5	

**Author**	**Control**	**Outcomes**	**Study period**	**Follow up**	**Treatment duration**	**Drop up**	**Registration number**	**Country**

Avdeev et al. (8)	Blank	The severity of symptoms, frequency of recurrence of COPD exacerbations, readmissions, need for emergency care and changes in basic therapy of COPD	NR	3 months	3 months	NR	NR	Russia
Zeng et al. (14)	Blank	The number of acute exacerbations per person per year, quality of life, lung function, T cell subsets	2015.07–2016.07	6 months	6 months	NR	NR	China
Tang et al. (9)	Placebo	The proportion of patients with recurrent acute exacerbations during the 12-week treatment period. Secondary efficacy endpoints included the proportion of patients with recurrent acute exacerbations over the 22-week study period, the proportion of patients treated	2005–2008	10 weeks	12 weeks	19 (4.9%)	China Food and Drug Administration TG0504BCV	China
Braido et al. (10)	Placebo	The primary outcome is the number of exacerbations, the secondary outcome is the time from the randomization to the first exacerbation, the average interval between the first and second exacerbation, the effects of Ismigen on symptoms	July 31, 2009, to June 16, 2012	3 months	3 months, 2 cycle	NR	EudraCT 2007-000006-67	Italy
Ricci et al. (13)	Placebo	Serological changes, exacerbation rate and symptom	Fall in 2019 to fall in 2010	About 3 months	3 months	0	NR	Italy
Nishantha et al. (15)	Placebo	Infective exacerbations and symptoms improvements	2012	6 months	NR	NR	NR	Sri Lanka
Olivieri (16)	Placebo	The rate and duration of AECOPD, the rate of treatment-emergent adverse events	2011	NR	NR	NR	NR	NR
Cazzola et al. (17)	Blank	Symptoms, diagnosis of exacerbation, concomitant medications and hospitalization	Begin from September 2007	3 months	3 months	0	NR	Italy
Solèr et al. (18)	Placebo	The primary endpoint was the mean rate of AEs occurring within the study period	NR	1 month	5 months	40 (14.65%)	NR	Switzerland and Germany
Li et al. (19)	Placebo	The frequency of acute exacerbation, symptom scores, and lung function were recorded	NR	9 months	3 month	0	NR	China
Collet et al. (20)	Placebo	The primary outcome was the occurrence of at least one such episode during the 6 months. Secondary outcomes included total number of acute exacerbations and hospitalization for a respiratory problem, as well as all hospitalization, change in baseline respiratory symptoms, and change in quality of life	Begin from November 07, 1994	2 months	4 months	15 (3.93%)	NR	Canada
Xinogalos et al. (21)	Placebo	Clinical manifestations, frequency, duration and severity of acute exacerbations, consumption of conventional medications and serum immunoglobulin levels	Autumn/winter of 1990–1991	2 months	4 months	–	–	–

*COPD, chronic obstructive pulmonary disease; GOLD, the global initiative for chronic obstructive lung disease; NR, not reported.*

**TABLE 2:** The characteristics of the included participants.

Author, year	Age (years) Mean [95% CI] or Mean ± SD		Gender (male/total)		Smoking history Mean ± SD		FEV1/predicted (%) Mean ± SD		Concomitant respiratory medications			Diabetes	
	Bacterial lysates	Control	Bacterial lysates	Control	Bacterial lysates	Control	Bacterial lysates	Control	Long-terms effect bronchodilator	Flu vaccination	Pneumonia vaccination	Bacterial lysates	Control
Avdeev et al. (8)	69.4 ± 9.4	69.9 ± 7.9	28/30	30/30	30/30	30/30	31.3 ± 8.7	33.2 ± 7.7	Yes	NR	NR	NR	NR
Zeng et al. (14)	70.1 ± 5.7	69.2 ± 6.1	67/78	63/72	63/78	61/72	49.15 ± 4.74	49.06 ± 2.73	Yes	No	No	NR	NR
Tang et al. (9)	63.0 ± 9.4	63.2 ± 8.9	141/192	133/192	123/192	120/192	54.0 ± 10.0	55.0 ± 9.7	Yes	Allowed	NO	NR	NR
Braido et al. (10)	69.3 ± 8.6	68.6 ± 9.4	107/146	91/142	123/146	120/142	52.18 ± 18.99	52.66 ± 16.26	Yes	Allowed	NR	19/146	10/142
Ricci et al. (13)	65–89		16/28		–	–	–	–	Yes	Allowed	NR	–	–
Nishantha et al. (15)	50–58		24/24	21/21	–	–	–	–	–	–	–	–	–
Cazzola et al. (17)	66.6 ± 7.8	66.2 ± 8.0	25/33	26/30	≥20/33	≥16/30	47.7 ± 9.0	46.2 ± 8.9	Yes	Allowed	NR	NR	NR
Solèr et al. (18)	57.3 [55.7, 58.9]	57.9 [56.2, 59.6]	78/142	57/131	87/142	77/131	85[81.7, 88.3]	82.6[79.9, 86.1]	Yes	Allowed	NR	NR	NR
Li et al. (19)	67 ± 4	65 ± 5	27/49	22/41	49/49	41/41	50.9 ± 21	53.2 ± 19.7	Yes (sustained- released theophylline)	NR	NR	NR	NR
Collet et al. (20)	65.3 ± 7.7	66.9 ± 7.7	133/191	135/190	78/191′	59/190′	67.7 ± 15.3	68.4 ± 15.6	NR	Allowed	NR	NR	NR
Xinogalos et al. (21)	56.03 ± 12.67	59.75 ± 12.89	–	–	–	–	–	–	–	–	–	–	–

*FEV1/predicted, forced expiratory volume in one second/predicted value; NR, not reported.*

### Quality of the Included Studies

**FIGURE 2:**
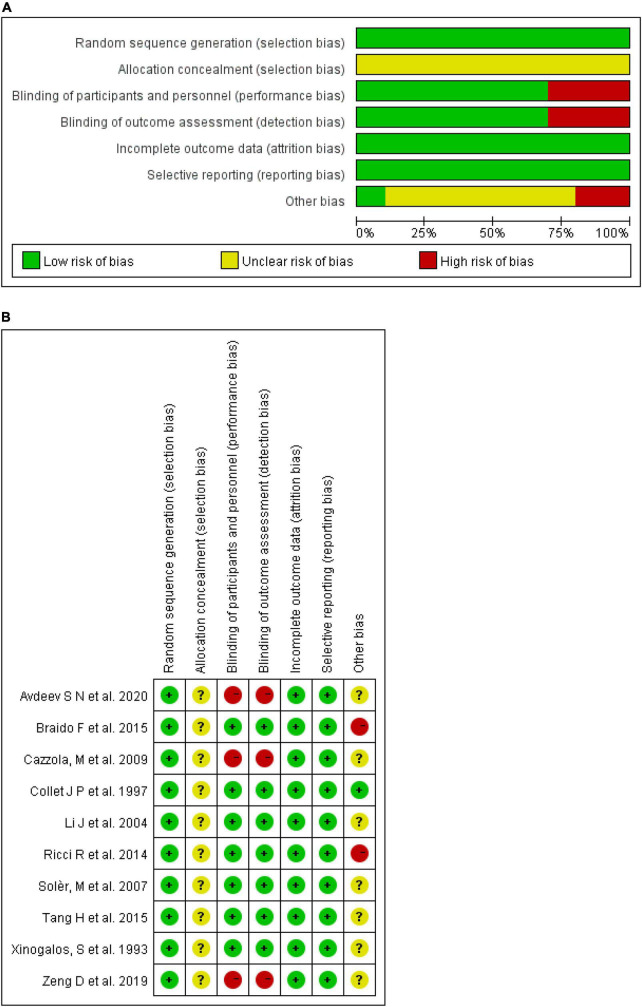
Risk of bias. **(A)** Each risk of bias item presented as percentages across all included studies. **(B)** Each risk of bias item for each included study.

Eligible studies with sufficient information were assessed using the Cochrane risk-of-bias tool. As shown in Figure 2, three studies with a blank control were at a high risk of both performance and detection bias. No study mentioned allocation concealment, which was deemed an unknown risk. Braido et al. (10) were funded by a pharmaceutical company and considered high risk. Tang et al. (9) were also sponsored by *OM Pharma* but all the authors assume responsibility for the integrity and completeness of the data and data analyses, and considered an unknown risk. The others did not include a *conflict of interest* section and were considered an unknown risk. There were low risks in random sequence generation, attrition bias, and reporting bias in all studies assessed. Figure 2 summarizes the quality of the included studies.

### Efficacy and Safety of Bacterial Lysates

#### Efficacy on Exacerbation

Nine studies reported the rate of exacerbation with detailed numbers. Excluding the study by Ricci et al. (13), data from eight studies were subtracted for the meta-analysis. The pooled result indicated that bacterial lysates could reduce the exacerbation rate by 17% (RR = 0.83, 95% CI 0.72–0.96, *P* = 0.01; heterogeneity: *P* = 0.02, *I*^2^ = 57%). The alkaline bacterial lysate (OM-85) subgroup reduced the exacerbation rate by 13% (RR = 0.87, 95% CI 0.77–0.98, *P* = 0.02; heterogeneity: *P* = 0.13, *I*^2^ = 44%), while the mechanical bacterial lysate (Ismigen) subgroup reduced the exacerbation rate by 30% (RR = 0.70, 95% CI 0.41–1.20, *P* = 0.19; heterogeneity: *P* = 0.01, *I*^2^ = 77%); however, the difference was not significant.

**FIGURE 3:**
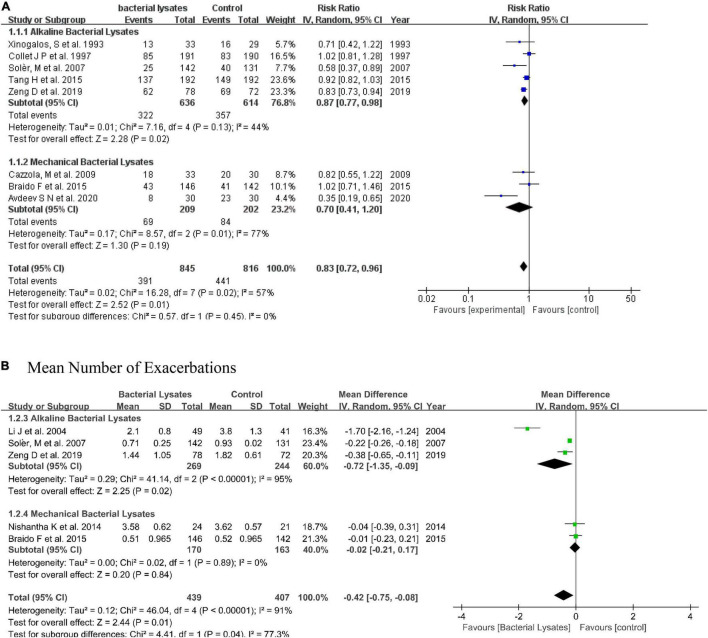
Forest plot of the efficacy of bacterial lysates on exacerbations. **(A)** Forest plot of rate of exacerbation. **(B)** Forest plot of mean number of exacerbations.

We also evaluated the efficacy of bacterial lysates on exacerbation using the mean number of exacerbations. In line with the rate of exacerbation, the pooled result showed that bacterial lysates were associated with fewer exacerbations (MD = −0.42, 95% CI −0.75 to −0.08, *P* = 0.01; heterogeneity: *P* < 0.001, *I*^2^ = 91%). Both the alkaline (MD = −0.72, 95% CI −1.35 to −0.09, *P* = 0.02; heterogeneity: *P* < 0.001, *I*^2^ = 95%) and mechanical (MD = −0.02, 95% CI −0.21 to 0.17, *P* = 0.20; heterogeneity: *P* = 0.89, *I*^2^ = 0) bacterial lysates decreased the mean number of exacerbations in the subgroup analysis. Another study also reported that mechanical bacterial lysates were effective at reducing exacerbations (22), but was excluded from this synthesis for lack of data. The forest plots are shown in Figure 3.

#### Efficacy on Hospitalization and Respiratory Infections

**FIGURE 4:**
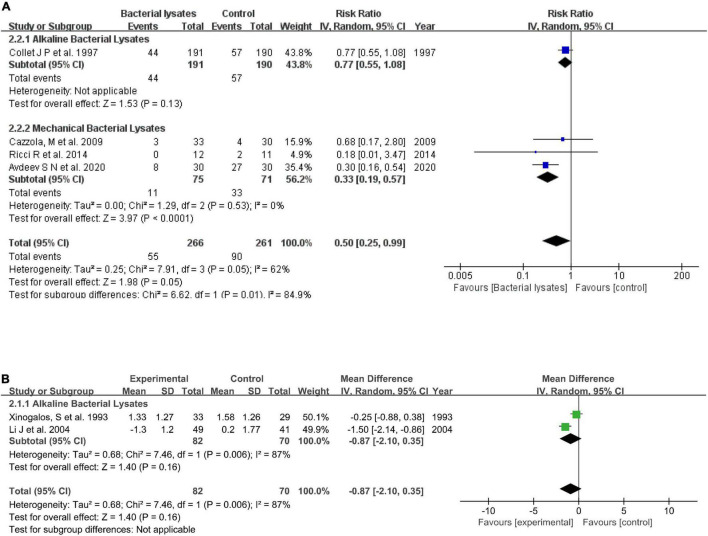
Forest plots of the efficacy of bacterial lysates on respiratory infections and hospitalization. **(A)** Rate of hospitalization. **(B)** Mean number of respiratory infections.

Four studies reported the hospitalization rate. Overall, bacterial lysates reduced hospitalization (RR = 0.50, 95% CI 0.25–0.99, *P* = 0.05; heterogeneity: *P* = 0.05, *I*^2^ = 62%). The subgroup analysis indicated that alkaline bacterial lysate treatment resulted in a 37% reduction in hospitalization (RR = 0.77, 95% CI 0.55–1.08, *P* = 0.13), while mechanical bacterial lysates resulted in a greater reduction in hospitalization (RR = 0.33, 95% CI 0.19–0.57, *P* < 0.001; heterogeneity: *P* = 0.52, *I*^2^ = 0). Moreover, Braido et al. (10) showed that mechanical bacterial lysates prolonged the interval between the first and second exacerbations (70.36 vs. 123.89 days, *P* = 0.03). Regarding the effect on respiratory infections, data from two studies showed the alkaline bacterial lysates induced an insignificant reduction in the mean number of respiratory infections (MD = −0.87, 95% CI −2.10 to 0.35, *P* = 0.16; heterogeneity: *P* = 0.006, *I*^2^ = 87%). Figure 4 shows the forest plots.

#### Efficacy on Symptoms

Symptoms were evaluated mainly using the changes in sputum, cough, severity of dyspnea, and fever.

Four studies reported a change in sputum, with two studies each examining alkaline and mechanical bacterial lysates. Collet et al. (20) described a difference between the OM-85 group and control without detailed data, while Li et al. (19) showed that alkaline bacterial lysates helped improve sputum (score 1.5 ± 0.5 vs. 1.9 ± 0.8, *P* < 0.01). Overall, bacterial lysates reduced the sputum slightly but insignificantly (SMD = −0.20, 95% CI −0.65 to 0.25, *P* = 0.39; heterogeneity: *P* = 0.08, *I*^2^ = 61%), while mechanical bacterial lysates had no significant effect on sputum (SMD = 0.04, 95% CI −0.32 to 0.41, *P* = 0.83; heterogeneity: *P* = 0.83, *I*^2^ = 0).

Five studies reported a change in cough. Collet et al. (20) (OM-85) and Solèr et al. (18) (Ismigen) also reported a difference between the two groups. Cazzola et al. (17) reported the same outcome (cough score 1.0 ± 0.6 vs. 1.0 ± 0.5). The remaining two eligible studies found OM-85 and improved cough. Data from the three studies for the meta-analysis gave an SMD of −0.46 for alkaline bacterial lysates (95% CI −0.72 to −0.21, *P* < 0.001; heterogeneity: *P* = 0.85, *I*^2^ = 0) and –0.37 for the overall effect of bacterial lysates (95% CI −0.61 to −0.14, *P* = 0.001; heterogeneity: *P* = 0.30, *I*^2^ = 18%).

**FIGURE 5:**
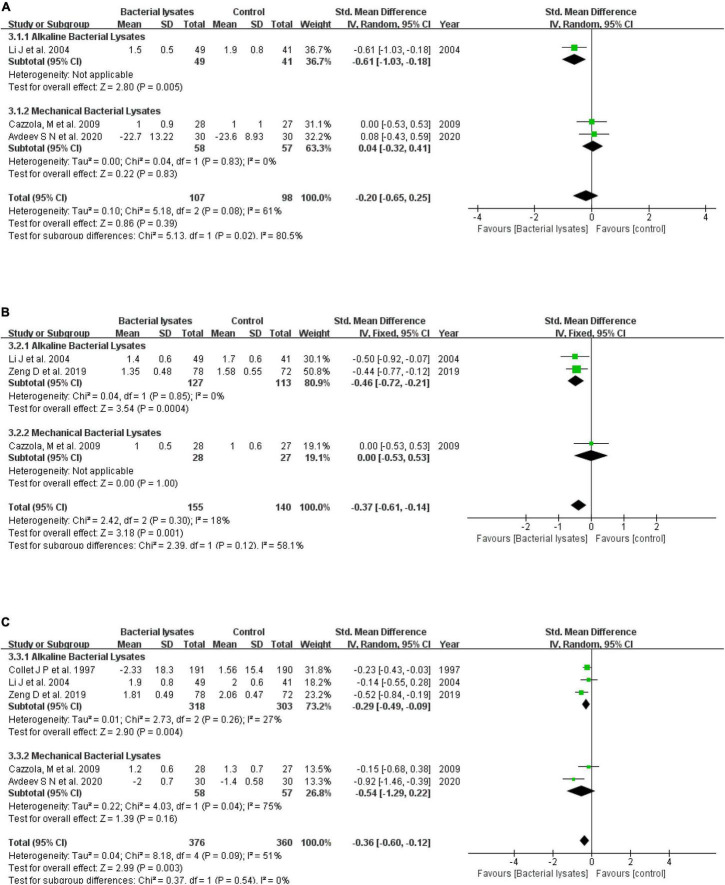
Forest plots of the efficacy of bacterial lysates on symptoms. **(A)** Sputum, **(B)** cough, and **(C)** severity of dyspnea.

Six studies reported a change in the severity of dyspnea. Solèr et al. (18) stated that there was no substantial difference between Ismigen and the control on dyspnea. Pooled results of the remaining five eligible studies indicated that bacterial lysates alleviated dyspnea (SMD = −0.36, 95% CI −0.60 to −0.12, *P* = 0.003; heterogeneity: *P* = 0.09, *I*^2^ = 51%). Both alkaline (SMD = −0.29, 95% CI −0.49 to −0.09, *P* = 0.004; heterogeneity: *P* = 0.26, *I*^2^ = 27%) and mechanical (SMD = −0.54, 95% CI −1.29 to 0.22, *P* = 0.16; heterogeneity: *P* = 0.04, *I*^2^ = 75%) bacterial lysates helped improve the severity of dyspnea, but a difference did not reach significance. Figure 5 shows the forest plots.

One study examined the effect of OM-85 on fever (10), reporting that the mean number of days with fever was 0.06 for the treatment group and 0.11 for the placebo group (*P* < 0.001).

#### Safety of Bacterial Lysates

**FIGURE 6:**
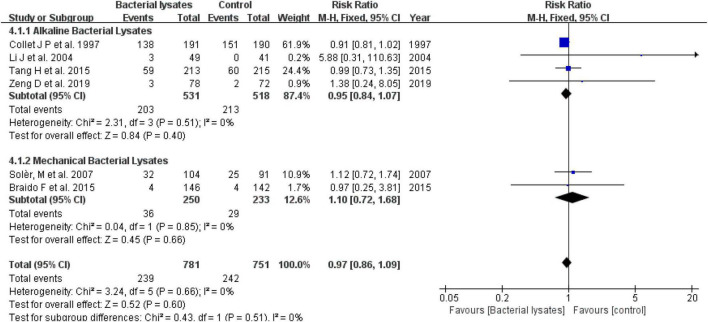
Safety of bacterial lysates: the forest plot of the rate of adverse events.

The difference in adverse effects between the bacterial lysates and control was similar and insignificant (RR = 0.97, 95% CI 0.86–1.09, *P* = 0.60; heterogeneity: *P* = 0.66, *I*^2^ = 0; Figure 6 shows the forest plot). The adverse effects reported in most studies were acceptable and slight. Table 3 summarizes the symptoms.

**TABLE 3:** The detailed adverse effect.

Author, year	Treatment	The most symptoms reported	
		Treatment group	Control group
Zeng et al. (14)	OM-85	Nausea, Thirst	Thirst
Tang et al. (9)	OM-85	Common cold, nausea, abdominal pain, headache	Common cold, headache, dizziness, nausea
Solèr et al. (18)	OM-85	Respiratory system manifestations such as acute exacerbations, tonsillitis, symptoms of influenza, sinusitis	
Li et al. (19)	OM-85	Dyspepsia, skin pruritus	Not mentioned
Collet et al. (20)	OM-85	Abdominal with gastroenteritis, miscellaneous, pulmonary and respiratory issues	Miscellaneous, pulmonary and respiratory issues, abdominal with gastroenteritis

### Heterogeneity Analysis

**FIGURE 7:**
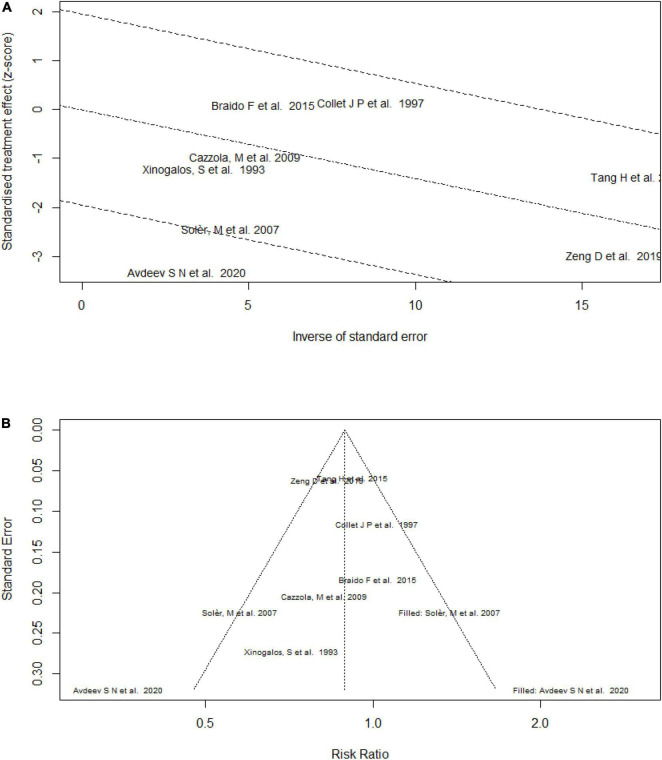
Radial plot and funnel plot of the efficacy of bacterial lysates on the rate of exacerbation. **(A)** Radial plot of the efficacy of bacterial lysates on the rate of exacerbation. **(B)** Funnel plot of the efficacy of bacterial lysates on the rate of exacerbation.

As the forest plots show, heterogeneity was present in the efficacy assessments. First, we conducted a subgroup analysis. The forest and radial plots (Figure 7) analyzing the effect on the exacerbation rate showed obvious heterogeneity for mechanical bacterial lysates; Avdeev et al. (8) included patients with a lower FEV_1_/predicted and found a higher RR, and might be the source of heterogeneity. After removing this study, the heterogeneity decreased (overall: RR = 0.88, 95% CI 0.79–0.97, *P* = 0.009; heterogeneity: *P* = 0.24, *I*^2^ = 25%; mechanical bacterial lysate subgroup: RR = 0.92, 95% CI 0.71–1.21, *P* = 0.57; heterogeneity: *P* = 0.42, *I*^2^ = 0). Examining the mean number of exacerbations, we noticed that the effect size differed among studies, but all supported the use of bacterial lysates. The heterogeneity decreased in the subgroups when analyzing the secondary efficacy outcomes.

Considering the limited number of studies (23), we also conducted a meta-regression of the rate of exacerbation. This suggested that the type of bacterial lysate (*P*-value of meta-regression 0.698) and publication year (*P*-value of meta-regression 0.727) was unlikely to be the cause of heterogeneity.

### Sensitivity Analysis

**FIGURE 8:**
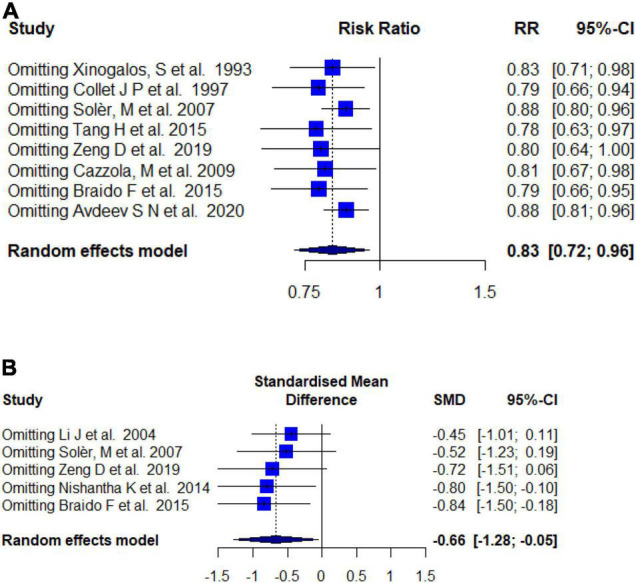
Sensitivity analysis. **(A)** Influence analysis of the rate of exacerbation. **(B)** Influence analysis of the mean number of exacerbations.

We performed a sensitivity analysis to evaluate the robustness of the efficacy results. A meta-analysis with an alternative model was used to assess whether the statistical method affected the results. As shown in Table 4, the value of the estimated effect was rather close. Moreover, we performed an influence analysis that examined the impact of each study on the final pooled effect size by re-synthesizing the included studies, by omitting one at a time (Figure 8). This indicated that the result remained stable no matter which study was removed.

**TABLE 4:** Results of the meta-analysis by the alternative model.

Outcome (effect factor)	Model used (estimated value [95% CI])	
	Random	Fixed
Rate of exacerbation (RR)	0.83 [0.72, 0.96]	0.87 [0.81, 0.93]
Mean number of exacerbations (MD)	−0.42 [−0.75, −0.08]	−0.23 [−0.27, −0.19]
Mean number of respiratory infections (MD)	−0.87 [−2.10, 0.35]	−0.87 [−1.32, −0.42]
Rate of hospitalization (RR)	0.50 [0.25, 0.99]	0.61 [0.46, 0.81]
Sputum (SMD)	−0.20 [−0.65, 0.25]	−0.23 [−0.51, 0.04]
Cough (SMD)	−0.37 [−0.63, −0.11]	−0.37 [−0.61, −0.14]
Severity of dyspnoea (SMD)	−0.36 [−0.60, −0.12]	−0.32 [−0.47, −0.18]
Adverse effects (RR)	0.93 [0.84, 1.03]	0.97 [0.86, 1.09]

### Publication Bias

Considering the limited number of included studies that were not suitable for statistical testing, the publication bias was assessed using a funnel plot. As shown in Figure 7, publication bias appeared to present, so a trim analysis was performed to help quantify its influence. However, with the addition of studies, it remained similar (RR = 0.88 [trim analysis] vs. RR = 0.83 [accrual]).

## Discussion

The use of bacterial lysates dates to the 1970s and they are most often used clinically to prevent or treat an infection. Evidence has accrued demonstrating their role in pediatric respiratory tract infections, and they are recommended in some guidelines (24). As they can be given orally, studies are examining their role in COPD.

Using published evidence, we evaluated the efficacy and safety of bacterial lysates in patients with COPD in this meta-analysis. This showed that bacterial lysates reduced exacerbations. This result was consistent with the former analysis by pan et al. (7) who included 4 randomized controlled trials focused on OM-85. The combined results of the RR in the OM-85 subgroup of our study and that of Pan et al. were 0.87 (95% CI 0.77–0.98) and 0.8 (95% CI 0.65–0.97), respectively. The results were also supported by several other studies that revealed that bacterial lysates were effective at reducing the exacerbations of chronic bronchitis (25, 26), which can lead to COPD. Several studies have linked the mechanism underlying the effects of lysates to the gut–lung axis. As oral immunomodulators, both mechanical and alkaline bacterial lysates interact with mucosa-associated lymphoid tissues in the gut, bronchi, and upper airways, which function as an integrated unit (27–29). On delivery to the body, the bacterial lysate antigens are captured and recognized by the pattern recognition receptors of immune cells in the mucosa, including dendritic cells. These then transmigrate to lymph nodes and bloodstream and trigger an immune cascade. As a result, secretory immunoglobulin A increases and there is an extensive non-specific immune response to pathogens and toxins (30–32). Moreover, bacterial lysates promote the production of antiviral cytokines, including INF-γ, facilitate neutrophil chemotaxis, and modulate the Th1/Th2 ratio, enhancing the immune status (31, 33–35). Consistent with this, fewer bacterial colonies were cultured in sputum from patients treated with bacterial lysates (19, 23), and less seroconversion was seen clinically (14).

Moreover, we assessed the efficacy of bacterial lysates on symptoms, which was not analyzed in the former meta-analysis. We found that bacterial lysates reduced the hospitalization rate, alleviated cough, and improved dyspnea slightly. There were also non-significant improvements in sputum production and the mean number of respiratory infections. Intriguingly, in the subgroup analysis, OM-85 was significantly more effective than Ismigen overall, although Ismigen might be more effective in reducing the hospitalization rate.

Of note, heterogeneity was seen in most results. This might have been due to the type of bacterial lysate, as seen in the subgroup analysis. In addition, the growing understanding of the disease and emerging drugs, which could be represented by the year, could also have influenced the outcomes. Thus, subsequent meta-regressions were performed. However, neither the type of bacterial lysate nor the year was a source of heterogeneity. Moreover, there was a publication bias in the rate of exacerbation, especially in the alkaline bacterial lysate subgroup. Nevertheless, the included studies all favored OM-85. In addition, it might be explained by differences in the population’s lung function, which was correlated with exacerbation risk. Importantly, a sensitivity analysis showed that our results were stable and reliable.

In summary, our results showed that bacterial lysates can reduce exacerbations and alleviate symptoms in patients with COPD.

Nevertheless, our study had several limitations. First, some studies did not report sufficient data in the abstract and were too old to obtain the full text, so they were removed from the meta-analysis. Second, the measurements in the studies, such as symptom evaluation, were not standardized, which could affect the quantitative analysis. In addition, several studies described the predefined outcomes rather than presenting detailed data and could not be included in the meta-analysis. Heterogeneity was also a problem, as discussed above. The included studies also had methodological issues, such as explicit allocation concealment, an important factor for evaluating selection bias, which could lead to bias and reduce the strength of the evidence.

## Conclusion

Bacterial lysates can benefit patients with COPD by reducing exacerbations and alleviating symptoms. OM-85 is the preferable product based on the existing evidence. Further studies are needed to validate these findings.

## Data Availability Statement

The original contributions presented in this study are included in the article/Supplementary Material, further inquiries can be directed to the corresponding author.

## Author Contributions

TZ provided study conception and funding support. YH designed search terms with the help of YP and YQ. YH completed the literature searching and mainly drafted the manuscript. YP and YQ finished study selection, quality assessment and revised the manuscript. YH and JD conducted quality assessment. MS did the data analysis and offered funding support. ZY and CC coped with the disagreement between the authors. All authors read and approved the final manuscript.

## Conflict of Interest

The authors declare that the research was conducted in the absence of any commercial or financial relationships that could be construed as a potential conflict of interest.

## Publisher’s Note

All claims expressed in this article are solely those of the authors and do not necessarily represent those of their affiliated organizations, or those of the publisher, the editors and the reviewers. Any product that may be evaluated in this article, or claim that may be made by its manufacturer, is not guaranteed or endorsed by the publisher.

## Supplementary Material

Supplementary Appendix 1 PRISMA checklist.

Supplementary Appendix 2 Lists the search terms.

## Abbreviations

COPD: chronic obstructive pulmonary disease
PRISMA: Preferred Reporting Items for Systematic Review and Meta-Analysis
SD: standard derivation
MD: mean difference
SMD: standard mean difference
CI: confidence interval
FEV1/predicted: forced expiratory volume in one second/predicted value
RR: relative risk.
