# Supplementary material for: A Meta-Analysis on the Efficacy and Safety of Bacterial Lysates in Chronic Obstructive Pulmonary Disease
Source: Front Med (Lausanne). 2022 Jun 9;9:877124. doi: 10.3389/fmed.2022.877124 (PMC9218542; doi:10.3389/fmed.2022.877124)
Supplement: Supplementary Appendix 1 — PRISMA checklist. [file Data_Sheet_1.pdf]

Pubmed

#1

"Pulmonary Disease, Chronic Obstructive"[Mesh] or "COPD" or "Chronic Obstructive Pulmonary Disease" or "COAD" or "Chronic Obstructive Airway Disease" or "Chronic Obstructive Lung Disease" or "Chronic Airflow Obstructions" or "Chronic Airflow Obstruction"

#2

“bacterial lysates” OR “bacterial lysate”

#3

“OM-85” OR ”Broncho-Vaxom” OR “OM-85BV” OR “Broncho Munal” OR “Ommunal” OR “Imoccur” OR ”Vaxoral” OR “Paxoral” OR “Ismigen” OR “Immubron” OR “Bromunyl” OR “Respibron” OR “Bactovax” OR “Ismigen PBML” OR “MV130” OR “Bactek” OR “Luivac LW50020” OR ”Luivac” OR “Lantigen B” OR ”Ribomunyl D53”

#4

#2 OR #3

#5

#1 AND #4

Embase

#1

'chronic obstructive lung disease'/exp

#2

'chronic airflow obstruction'

#3

'chronic airflow obstruction'

#4

'chronic obstructive airway disease'

#5

'chronic obstructive pulmonary disease'

#6

'COPD'

#7

'COAD'

#8

#1 OR #2 OR #3 OR #4 OR #5 OR #6 OR #7

#9

'bacterial lysates'

#10

'bacterial lysate'

#11

'bacterium lysate'

#12

'bacterium lysates'

#13

'OM-85' OR 'Broncho-Vaxom' OR 'OM-85BV' OR 'Broncho Munal' OR 'Ommunal' OR 'Imoccur' OR 'Vaxoral' OR 'Paxoral' OR 'Ismigen' OR 'Immubron' OR 'Bromunyl' OR 'Respibron' OR 'Bactovax' OR 'Ismigen PBML' OR 'MV130' OR 'Bactek' OR 'Luivac LW50020' OR 'Luivac' OR 'Lantigen B' OR 'Ribomunyl D53'

#14

#9 OR #10 OR #11 OR #12 OR #13

#15

#8 AND #14

#16

#15 AND [humans]/lim

the Cochrane Library (central)

#1

MeSH descriptor: [Pulmonary Disease, Chronic Obstructive] explode all trees

#2

"COPD" or "Chronic Obstructive Pulmonary Disease" or "COAD" or "Chronic Obstructive Airway Disease" or "Chronic Obstructive Lung Disease" or "Chronic Airflow Obstructions" or "Chronic Airflow Obstruction"

#3

#1 OR #2

#4

bacterial lysates

#5

bacterial lysate

#6

“OM-85” OR ”Broncho-Vaxom” OR “OM-85BV” OR “Broncho Munal” OR “Ommunal” OR “Imoccur” OR ”Vaxoral” OR “Paxoral” OR “Ismigen” OR “Immubron” OR “Bromunyl” OR “Respibron” OR “Bactovax” OR “Ismigen PBML” OR “MV130” OR “Bactek” OR “Luivac LW50020” OR ”Luivac” OR “Lantigen B” OR ”Ribomunyl D53”

#7

#4 OR #5 OR #6

#8

#3 AND #7

Web of Science

#1

((TS=(Chronic Obstructive Pulmonary Disease)) OR TS=(COPD)) OR TS=(Chronic Obstructive Airway Disease)

#2

((AB=(Chronic Obstructive Pulmonary Disease)) OR AB=(COPD)) OR AB=(Chronic Obstructive Airway Disease)

#3

((TI=(Chronic Obstructive Pulmonary Disease)) OR TI=(COPD)) OR TI=(Chronic Obstructive Airway Disease)

#4

#1 OR #2 OR #3

#5

(((((((((((((TI=(bacterial lysates)) OR TI=(OM-85)) OR TI=(Broncho-Vaxom)) OR TI=(OM-85BV)) OR TI=(Broncho Munal)) OR TI=(Ommunal)) OR TI=(Imoccur)) OR TI=(Vaxoral)) OR TI=(Ismigen)) OR TI=(Immubron)) OR TI=(Ismigen PBML)) OR TI=(Lantigen)) OR TI=(Ribomunyl)) OR TI=(Luivac)) OR TI=(Bactovax)

#6

(((((((((((((TS=(bacterial lysates)) OR TS=(OM-85)) OR TS=(Broncho-Vaxom)) OR TS=(OM-85BV)) OR TS=(Broncho Munal)) OR TS=(Ommunal)) OR TS=(Imoccur)) OR TS=(Vaxoral)) OR TS=(Ismigen)) OR TS=(Immubron)) OR TS=(Ismigen PBML)) OR TS=(LanTSgen)) OR TS=(Ribomunyl)) OR TS=(Luivac)) OR TS=(Bactovax)

#7

(((((((((((((AB=(bacterial lysates)) OR AB=(OM-85)) OR AB=(Broncho-Vaxom)) OR AB=(OM-85BV)) OR AB=(Broncho Munal)) OR AB=(Ommunal)) OR AB=(Imoccur)) OR AB=(Vaxoral)) OR AB=(Ismigen)) OR AB=(Immubron)) OR AB=(Ismigen PBML)) OR AB=(LanABgen)) OR AB=(Ribomunyl)) OR AB=(Luivac)) OR AB=(Bactovax)

#8

#4 AND #7
